# Early developmental changes in GABAA receptor expression in nucleus accumbens medium spiny neurons

**DOI:** 10.3389/fnins.2024.1445162

**Published:** 2024-12-12

**Authors:** Ziyi (Zephyr) Wang, Oluwarotimi O. Folorunso, Kiely Morris, Sabina Berretta, Elif Engin

**Affiliations:** ^1^Stress Neurobiology Laboratory, Division of Basic Neuroscience, McLean Hospital, Belmont, MA, United States; ^2^Department of Psychiatry, Harvard Medical School, Boston, MA, United States; ^3^Translational Neuroscience Laboratory, Division of Basic Neuroscience, McLean Hospital, Belmont, MA, United States

**Keywords:** nucleus accumbens, striatum, medium spiny neuron, neurodevelopment, GABAA receptor, GABA, *in situ* hybridization

## Abstract

The expression of GABA_A_Rs goes through large scale, evolutionarily conserved changes through the early postnatal period. While these changes have been well-studied in brain regions such as the hippocampus and sensory cortices, less is known about early developmental changes in other brain areas. The nucleus accumbens (NAc) is a major hub in the circuitry that mediates motivated behaviors and disruptions in NAc activity is a part of the neuropathology observed in mood and substance use disorders. Considering the importance of early developmental disruptions in the vulnerability to and etiology of these disorders, it is essential to understand normal developmental changes in the NAc as a first step to understanding how these changes might be disrupted to cause long-term pathology. Here, we aimed to address the gap in knowledge of early developmental changes in GABA_A_R expression in NAc neurons. We investigated the expression patterns of GABA_A_R α1, α2, and α4 subunits in Drd1+, Drd2+, and putative hybrid medium spiny neurons (MSNs) of the mouse NAc over a developmental window from P2 to P16. Our findings show a consistent increase in expression of all 3 GABA_A_R subunits in Drd1+ MSNs, accompanied by stable expression or even a decrease in expression in Drd2+ MSNs. The putative hybrid population showed a complex expression pattern, usually showing maximum expression at P9. These early developmental changes likely suggest a specific window where GABA_A_R expression patterns adjust to increasing glutamatergic inputs from external sources, changes in intracellular chloride concentrations, and a switch towards the mature, bistable activity patterns of MSNs from the immature, relatively excitable singular pattern. We propose that this time of dynamic changes in GABA_A_R expression could represent a sensitive period during which developmental insults might lead to permanent disruptions in GABA_A_R expression patterns.

## 1 Introduction

The nucleus accumbens (NAc) plays pivotal roles in reward-related and motivational processes, as well as in negative valence states (Klawonn and Malenka, 2018). Disrupted NAc activity is a hallmark of mood and substance use disorders (Fox and Lobo, 2019; Russo and Nestler, 2013; Suckling and Nestor, 2017), the etiology of which is deeply rooted in early developmental processes (Levis et al., 2022; Liu, 2017; McCrory and Mayes, 2015; Smith and Pollak, 2020).

NAc is composed of GABAergic projection neurons known as medium spiny neurons (MSNs, ~95% of NAc neurons) and GABAergic and cholinergic interneurons. The MSNs of the NAc are typically classified into two populations based on the expression of dopamine D1 (Drd1+ MSNs) or D2 (Drd2+ MSNs) receptors (Gerfen, 1992; Kreitzer, 2009). These two MSN subpopulations are integrated into different macrocircuits in the brain, serve different and often opposing functions in shaping emotional and motivational behaviors, and are influenced differentially by manipulations such as chronic stress and early life adversity (Francis et al., 2015; Gerfen, 1992; Hikida et al., 2010; Kronman et al., 2021; Kupchik et al., 2015; Lobo et al., 2010; McCullough et al., 2021). Under normal conditions, the activity of these two subpopulations of MSNs is balanced to support behavioral output that promotes the well-being of the organism. A bias for one subpopulation over the other, on the other hand, might lead to behavioral disturbances reminiscent of the behavioral pathology of mood and/or substance use disorders (Fox and Lobo, 2019; Francis and Lobo, 2017; Kim et al., 2009; Lee et al., 2006; Russo et al., 2010).

The activity of NAc MSNs is strictly regulated by NAc interneurons as well as via collateral inhibitory transmission between MSNs (Tunstall et al., 2002; Tepper et al., 2004), which supports the above-noted Drd1+ − Drd2+ MSN balance and the precise activity of the brain circuits these neuronal subpopulations are a part of. The effect of GABAergic interneurons and collateral inhibition on MSN activity is mediated by the diverse members of the GABA_A_ receptor (GABA_A_R) family expressed on these neurons. The diversity of GABA_A_R-mediated neurotransmission stems from the numerous configurations that can potentially be assumed by these pentameric complexes formed through combinations drawn from16 potential subunit types (in addition to the *ρ* subunit which does not co-assemble with the other subunits), although most of the possible combinations are not observed in the brain (Rudolph and Knoflach, 2011; Engin et al., 2018). Further simplification is afforded by the common colloquialism of classifying GABA_A_Rs into subtypes based on the *α* subunit they carry, as this subunit dictates receptor properties and influences other subunits expressed in the assembly. Three GABA_A_R subtypes, α1, α2, and α4-containing GABA_A_Rs (α1GABA_A_Rs, α2GABA_A_Rs, α4GABA_A_Rs) are expressed prominently in the adult rodent NAc (Fritschy and Mohler, 1995; Pirker et al., 2000; Hortnagl et al., 2013). An additional subtype, α5GABA_A_Rs, is observed heavily in the primate NAc but is not abundant in the rodent NAc (Sperk et al., 2020). The subunit composition of the GABA_A_Rs determines receptor kinetics and subcellular location, dictating the ultimate impact of receptor activation on the affected neuron, allowing for complex and nuanced control of MSN activity.

During prenatal and early postnatal development, the expression of different GABA_A_Rs goes through extensive changes following predictable patterns. These patterns are conserved across species as diverse as zebrafish, pigs, rodents, nonhuman primates, and humans, suggesting an essential developmental role (Monesson-Olson et al., 2018; Laurie et al., 1992; Miller et al., 2014, 2017; Duncan et al., 2010). For instance, α1GABA_A_Rs, which are the predominant subtype in the adult, are expressed at low levels prenatally and their expression increases gradually starting shortly before birth and continuing postnatally, while α2GABA_A_Rs follow the opposite pattern (Fritschy et al., 1994; Laurie et al., 1992; van Eden et al., 1995; Hornung and Fritschy, 1996). In rats, the switch from α2 to α1 dominance takes place around postnatal day 5 (P5), while in humans, it is likely within the second year of life (Duncan et al., 2010). In areas of high expression, such as the thalamus, α4GABA_A_R expression shows a steady increase from late embryonic through postnatal development in rats, with more constant expression patterns starting at early postnatal development in other areas (Laurie et al., 1992). The postnatal expression of GABRA4 in the human cortex seems to follow a slightly different pattern, with expression reaching a peak within the first year of life and showing gradual decline afterwards (Duncan et al., 2010). Studies suggest that early life insults that take place during this time of dynamic shifts in GABA_A_R expression can lead to a disruption in this process, leading to an immature, α2-dominant expression pattern in at least some brain areas, with consequences for neuronal activity and behavioral output (Hsu et al., 2003). Similarly, experimental disruption of normal expression patterns of GABA_A_R subtypes during these sensitive developmental windows has large-scale repercussions for brain function and behavior (Topchiy et al., 2024), suggesting important roles for this pattern of changes in GABA_A_R expression and sensitivity of these patterns to external events.

Most of our knowledge regarding changes in GABA_A_Rs during early development comes from the hippocampus and prefrontal cortex, however, there is some evidence of similar GABA_A_R expression changes taking place in the striatum (Laurie et al., 1992). There have, however, been no studies focusing specifically on the NAc to our knowledge. With some work suggesting differences between dorsal striatal and NAc MSNs in terms of their activity patterns and eventual projection targets (e.g., Kupchik et al., 2015), it is not clear whether developmental findings from the dorsal striatum can safely be generalized to the NAc. Moreover, as noted above, Drd1+ and Drd2+ MSNs play different and often opposing roles in shaping behavioral output and the delicate balance between the activity of the two NAc subpopulations seems to be essential for maintaining normal, adaptive behavioral output, with biases in favor of either subpopulation potentially leading to behavioral pathology. Finally, early developmental processes, including developmental insults, seem to play an integral role in both adaptive NAc function in adolescence and adulthood (e.g., Goff et al., 2013; Wang et al., 2024) and in the healthy maturation of GABA_A_R expression patterns (Topchiy et al., 2024). As such, an understanding of the baseline developmental patterns of GABA_A_R-mediated inhibitory control of Drd1+ and Drd2+ MSNs is fundamental for the study of the normal development of the NAc and the contribution of early developmental processes and early experiences to NAc dysfunction that eventually contributes to the development of behavioral pathology in mood and substance use disorders.

Here, we aim to address this gap in knowledge by mapping the expression patterns of mRNA for the three common GABA_A_R subtypes, α1, α2, and α4GABA_A_Rs, in the Drd1+ and Drd2+ MSNs of the NAc during early postnatal development in mice. We additionally focus on a third population expressing both Drd1 and Drd2 (Drd1+/Drd2+), possibly representing a recently identified hybrid or atypical MSN population with different characteristics (Gagnon et al., 2017; Saunders et al., 2018; Stanley et al., 2020; He et al., 2021). Our findings suggest dynamic and distinct changes of GABA_A_R mRNA expression in the three subpopulations. Of particular significance is the finding that the expression levels of the same subunit RNA often change in opposite directions in the Drd1+ and Drd2+ MSN subpopulations with maturation such that the overall expression of the subunit RNA remains relatively stable over time when the tissue is analyzed globally, emphasizing the importance of cell-type-specific investigation for an accurate depiction of developmental changes.

## 2 Materials and methods

### 2.1 Animals

Male offspring of C57BL6/J mice (Jackson Laboratories, Bar Harbor, ME) bred in-house at McLean Hospital Animal Care Facility were used in the studies. Male–female pairs of mice were housed in polycarbonate cages and were maintained on a 12-h light–dark cycle (lights on: 0700) with food and water available *ad libitum*. The male was removed from the cage once pregnancy was detected. All procedures were performed in accordance with the National Institutes of Health guidelines, ARRIVE guidelines, and in compliance with the policies of McLean Hospital Institutional Animal Care and Use Committee.

### 2.2 Tissue preparation

The offspring were removed from breeding cages on P2, P9, or P16 and were administered an overdose (200 mg/kg, i.p.) of pentobarbital (Millipore Sigma, Burlington, MA), or 5% isoflurane prior to transcardial perfusion with sodium phosphate buffer followed by 4% paraformaldehyde. The brains were removed quickly and were kept in a post-fix of 4% paraformaldehyde at 4°C for 24 h. Subsequently, they were transferred to 20% followed by 30% sucrose solutions for cryoprotection. The sucrose cryoprotected brains were cut into 14 μm thick sections and were mounted on gelatin-coated slides for *in situ* hybridization.

### 2.3 Fluorescent *in situ* hybridization (RNAscope)

RNAscope (Wang et al., 2012) *in situ* hybridization was performed using the RNA Scope Multiplex Fluorescent Reagent Kit v2 (ACD Biosciences, cat# 323100) according to the manufacturer’s specifications (ACD Bioscience, RNAscope Multiplex Fluorescent v2 Assay Manual, 323100-USM). The following probes were used: Mm-Gabra1 (cat#435351), Mm-Gabra2 (cat#435011), Mm-Gabra4 (cat#424261), Mm-Drd1-C2 (cat#461901), Mm-Drd2-C3 (cat#406501). Opal fluorophores were used for visualization, with Opal 520 (cat# FP1487001KT) for Gabra1, Gabra2, and Gabra4, Opal 570 (cat# FP1488001KT) for Drd1, and Opal 690 (cat# FP1497001KT) for Drd2. The dilution for each probe was 1:1000, 1:1000, 1:1500, 1:1000, and 1:1000, respectively. We included both positive and negative controls in each RNAscope run for quality control and background signal determination.

### 2.4 Quantification

The number of Gabra1+, Gabra2+, Gabra4+, Drd1+, Drd2+ neurons was estimated using Z-stack images obtained on a Leica SP8 confocal microscope from four consecutive coronal sections that included the NAc for each animal (*N* = 4 animals per developmental timepoint; see Supplementary Figure S1 for a summary of the experimental procedures). The images were exported and analyzed using HALO imaging analysis software (ISH Quantification Module; Indica Labs). The region of interest (ROI), specifically the NAc, was identified referring to Allen Brain Atlas: mouse brain and developing mouse brain (see Supplementary Figures S1, S2). For the Developing Mouse Brain atlas sections, the sections with the closest timepoint, (i.e., P7 for the P9 brains and P14 for the P16 brains) were used, as no reference figures exist for the P9 and P16 timepoints. Anterior commissure and the ventral tip of the lateral ventricle were used as reference points while marking the ROI. Drd2 staining was used as a secondary *post hoc* guide to confirm striatal boundaries, as this target is expressed at high levels in the striatum but at negligeable levels in neighboring areas.

Signal detection and quantification were restricted to the annotated NAc area. We used AI custom detection settings, and adjusted minimum intensity, segmentation threshold for each image to account for variations in DAPI staining brightness. Overall number of cells in NAc was estimated by counting the number of DAPI+ nuclei from all Z-stacks for each animal. The total proportion of cells expressing a specific target (e.g., proportion of Drd1+ cells) was calculated as the number of target positive nuclei divided by the number of DAPI nuclei. The populations of Drd1+, Drd2+, and Drd1+/Drd2+ MSNs, on the other hand, were defined as cells that express Drd1 but not Drd2, cells that express Drd2 but not Drd1, and cells that express both Drd1 and Drd2, respectively.

### 2.5 Statistical analysis

Statistics were calculated using Prism 10 (GraphPad Software, San Diego, CA, USA). For overall analyses of GABA_A_R subunit or dopamine receptor expression, one-way ANOVA with developmental time-points (P2, P9, P16) as the levels was used, followed by Tukey’s *post hoc* tests where a statistically significant difference between levels was observed in the ANOVA. For cell-type-specific analysis of GABA_A_R subunit RNA expression across developmental time-points, two-way ANOVAs with cell-type (Drd1+, Drd2+, Drd1+/Drd2+) and developmental time-point (P2, P9, P16) as factors were used separately for each GABA_A_R subunit (Gabra1, Gabra2, Gabra4). Where the initial two-way ANOVA yielded a statistically significant factor, a Tukey’s *post hoc* test was used for multiple comparisons. For convenience, we have summarized the results of the statistical tests in Tables 1, 2. In figures, data are presented as mean ± SEM. *p < 0.05, **p < 0.01, ***p < 0.001.

**Table 1:** Statistical comparisons (One-Way ANOVA) for overall mRNA expression of the studied targets between different developmental timepoints.

Gene	*F*-value (ANOVA)	*p*-value (ANOVA)	Summary	Multiple comparisons	Mean diff.	95.00% CI of diff.	*p*-value (*post hoc*)	Summary
Drd1	*F* (2, 9) = 19.30	*p* = 0.0006	***	P2 vs. P9	−21.48	−31.15 to −11.82	0.0004	***
				P2 vs. P16	−9.8	−19.47 to −0.1330	0.0471	*
				P9 vs. P16	11.68	2.015 to 21.35	0.0202	*
Drd2	*F*(2, 9) = 8.370	*p* = 0.0088	**	P2 vs. P9	0.6561	−7.813 to 9.125	0.9746	ns
				P2 vs. P16	11.06	2.592 to 19.53	0.0133	*
				P9 vs. P16	10.41	1.936 to 18.87	0.0186	*
Gabra1	*F*(2, 9) = 4.380	*p* = 0.0469	*	P2 vs. P9	−10.15	−22.59 to 2.289	0.1107	ns
				P2 vs. P16	−12.36	−24.80 to 0.07342	0.0513	ns
				P9 vs. P16	−2.215	−14.65 to 10.22	0.8744	ns
Gabra2	*F*(2, 9) = 0.07549	*p* = 0.9279	ns	P2 vs. P9	−1.249	−12.04 to 9.542	0.9444	ns
				P2 vs. P16	−1.347	−12.14 to 9.445	0.9358	ns
				P9 vs. P16	−0.09728	−10.89 to 10.69	0.9997	ns
Gabra4	*F*(2, 9) = 76.99	*p* < 0.0001	****	P2 vs. P9	−38.89	−49.17 to −28.60	<0.0001	****
				P2 vs. P16	−40.26	−50.55 to −29.98	<0.0001	****
				P9 vs. P16	−1.379	−11.66 to 8.907	0.9263	ns

The left column presents the results of the initial ANOVA with timepoint (P2, P9, P16) as the factor. The right column presents the results of the Tukey’s *post hoc* test with comparisons between each timepoint. *p<0.05, **p<0.01, ***p<0.001, ****p<0.0001.

**Table 2:** Statistical comparisons (two-way ANOVA) for cell-type specific mRNA expression of the studied GABA_A_R subunits at different developmental timepoints.

Gene—source of variation	*F*-value (ANOVA)	*p*-value (ANOVA)	Summary	Multiple comparsions	Mean diff.	*P*-value (*post hoc*)	Summary
Gabra1							
Cell type × Timepoint	*F*(4, 18) = 28.17	*p* < 0.0001	****	Within Drd1+			
Cell type	F (2, 9) = 32.37	*p* < 0.0001	****	P2 vs. P9	−9.499	0.0073	**
Timepoint	*F* (2, 18) = 6.001	*p* = 0.0101	*	P2 vs. P16	−20.17	<0.0001	****
				P9 vs. P16	−10.67	0.0029	**
				Within Drd2+			
				P2 vs. P9	12.18	0.0009	***
				P2 vs. P16	5.71	0.1211	ns
				P9 vs. P16	−6.471	0.0723	ns
				Within Drd1+/Drd2+			
				P2 vs. P9	−16.8	<0.0001	****
				P2 vs. P16	0.1182	0.999	ns
				P9 vs. P16	16.92	<0.0001	****
				Within P2			
				Drd1 vs. Drd2	−11.37	0.0013	**
				Drd1+ vs. Drd1+ Drd2+	−13.73	0.0001	***
				Drd2+ vs. Drd1+ Drd2+	−2.367	0.6871	ns
				Within P9			
				Drd1 vs. Drd2	10.32	0.0033	**
				Drd1+ vs. Drd1+ Drd2+	−21.03	<0.0001	****
				Drd2+ vs. Drd1+ Drd2+	−31.35	<0.0001	****
				Within P16			
				Drd1 vs. Drd2	14.51	<0.0001	****
				Drd1+ vs. Drd1+ Drd2+	6.554	0.0726	ns
				Drd2+ vs. Drd1+ Drd2+	−7.959	0.0247	*
Gabra2							
Cell type × Timepoint	F (4, 18) = 5.282	*p* = 0.0054	**	Within Drd1+			
Cell type	F (2, 9) = 18.56	P = 0.0006	***	P2 vs. P9	−14.89	0.0224	*
Timepoint	F (2, 18) = 0.3796	*p* = 0.6895	ns	P2 vs. P16	−16.38	0.012	*
				P9 vs. P16	−1.494	0.9531	ns
				Within Drd2+			
				P2 vs. P9	10.69	0.1148	ns
				P2 vs. P16	5.806	0.4976	ns
				P9 vs. P16	−4.881	0.607	ns
				Within Drd1+ Drd2+			
				P2 vs. P9	−3.413	0.7805	ns
				P2 vs. P16	7.151	0.3543	ns
				P9 vs. P16	10.56	0.1199	ns
				Within P2			
				Drd1 vs. Drd2	−5.106	0.5442	ns
				Drd1+ vs. Drd1+ Drd2+	−15.2	0.0103	*
				Drd2+ vs. Drd1+ Drd2+	−10.09	0.1081	ns
				Within P9			
				Drd1 vs. Drd2	20.47	0.0006	***
				Drd1+ vs. Drd1+ Drd2+	−3.726	0.7205	ns
				Drd2+ vs. Drd1+ Drd2+	−24.19	<0.0001	****
				Within P16			
				Drd1 vs. Drd2	17.08	0.0039	**
				Drd1+ vs. Drd1+ Drd2+	8.332	0.2106	ns
				Drd2+ vs. Drd1+ Drd2+	−8.748	0.1814	ns
Gabra4							
Cell type × Timepoint	F (4, 18) = 7.712	*p* = 0.0008	***	Within Drd1+			
Cell type	F (2, 9) = 50.26	P < 0.0001	****	P2 vs. P9	−19.63	0.0011	**
Timepoint	F (2, 18) = 14.63	*p* = 0.0002	***	P2 vs. P16	−26.15	<0.0001	****
				P9 vs. P16	−6.517	0.3414	ns
				Within Drd2+			
				P2 vs. P9	1.711	0.9243	ns
				P2 vs. P16	−5.671	0.4378	ns
				P9 vs. P16	−7.382	0.2577	ns
				Within Drd1+ Drd2+			
				P2 vs. P9	−19.56	0.0011	**
				P2 vs. P16	−3.997	0.6567	ns
				P9 vs. P16	15.56	0.0078	**
				Within P2			
				Drd1 vs. Drd2	−5.729	0.3699	ns
				Drd1+ vs. Drd1+ Drd2+	−18.78	0.0003	***
				Drd2+ vs. Drd1+ Drd2+	−13.05	0.0115	*
				Within P9			
				Drd1 vs. Drd2	15.61	0.0025	**
				Drd1+ vs. Drd1+ Drd2+	−18.7	0.0004	***
				Drd2+ vs. Drd1+ Drd2+	−34.32	<0.0001	****
				Within P16			
				Drd1 vs. Drd2	14.75	0.0042	**
				Drd1+ vs. Drd1+ Drd2+	3.376	0.7015	ns
				Drd2+ vs. Drd1+ Drd2+	−11.37	0.0293	*

The left column presents the results of the initial ANOVA with cell-type (Drd1+, Drd2+, Drd1+/Drd2+) and timepoint (P2, P9, P16) as the factors. The right column presents the results of the Tukey’s *post hoc* test with comparisons within each level of the factors. *p<0.05, **p<0.01, ***p<0.001, ****p<0.0001.

## 3 Results

### 3.1 Developmental changes in the overall proportion of Drd1 and Drd2 expressing cells

**Figure 1:**
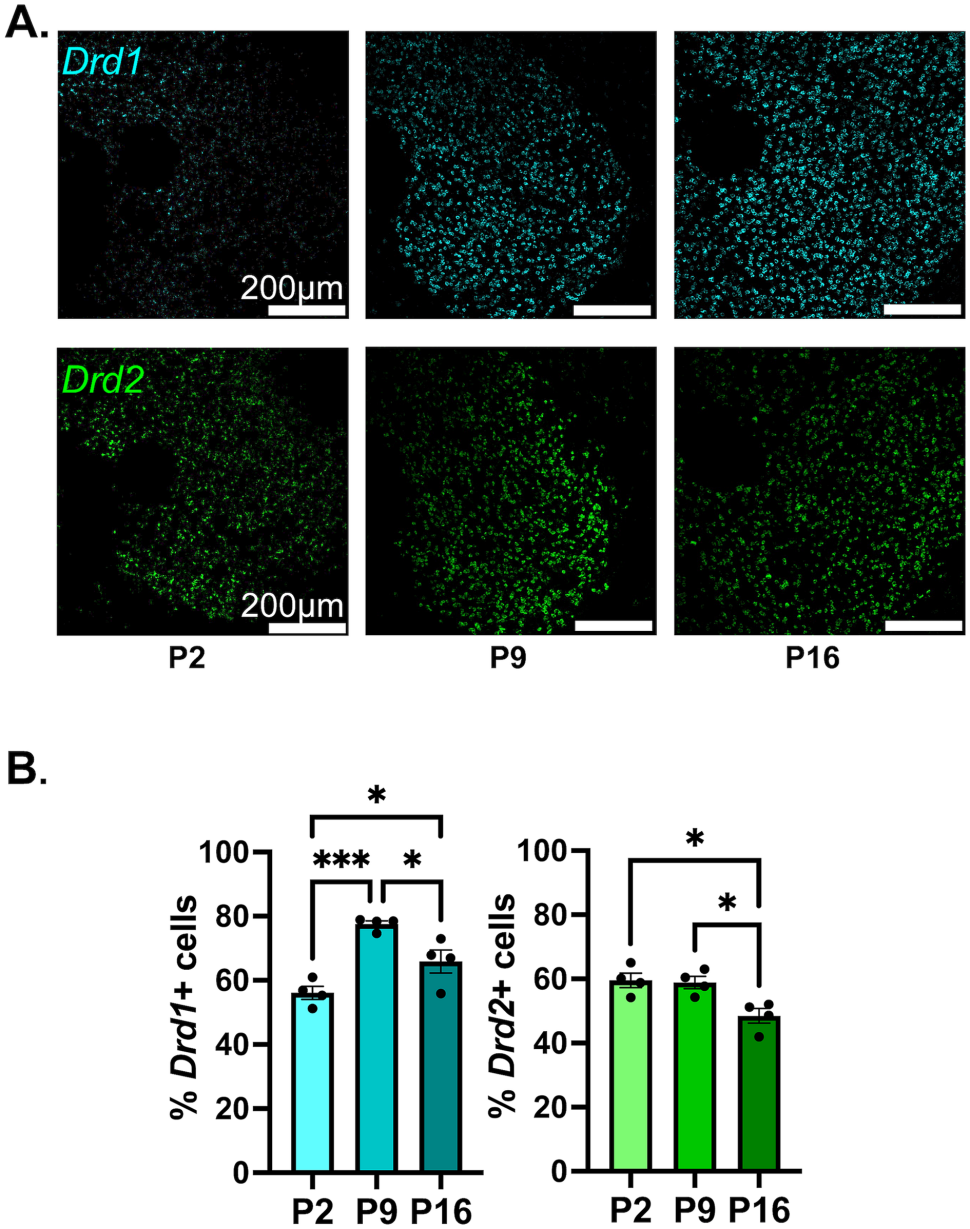
Developmental changes in the overall percentage of NAc cells expressing Drd1 or Drd2. **(A)** Representative confocal microscopy images of sections from P2, P9, and P16 mice probed for Drd1 (top) and Drd2 (bottom). **(B)** Quantification of percentage of cells expressing Drd1 (left) and Drd2 (right) out of all MSN cells (dapi+ nuclei). *p < 0.05, ***p < 0.001.

We observed a significant increase in the proportion of NAc cells expressing Drd1 from P2 to P9 and P16, with the highest proportion (~80%) observed at the P9 timepoint (Figures 1A,B; see Table 1, top section for a summary of the statistical test results). The overall proportion of NAc cells expressing Drd2 followed an opposite trend, with the highest proportions (~60%) observed at the P2 timepoint and a significant reduction from this by the P16 timepoint. At P2, the percentage of Drd1 expressing and the percentage of Drd2 expressing cells appear to be almost equal, with a slight advantage for Drd2, whereas by P16, there is clear Drd1 dominance.

### 3.2 Developmental changes in the overall proportion of Gabra1, Gabra2, or Gabra4 expressing cells

**Figure 2:**
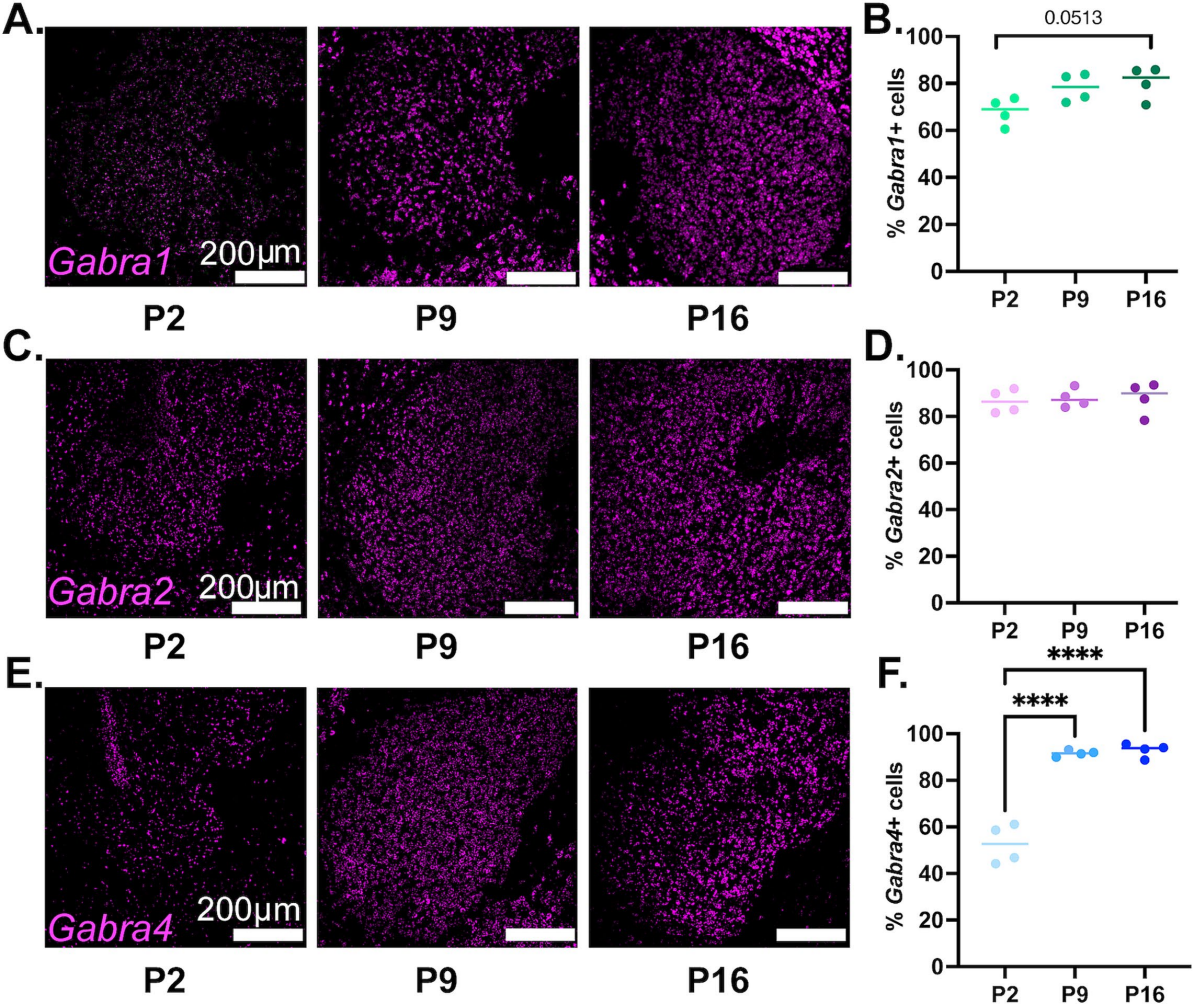
Developmental changes in the overall percentage of NAc cells expressing Gabra1, Gabra2, or Gabra4. **(A,C,E)** Representative confocal microscopy images of sections from P2, P9, and P16 mice probed for Gabra1 **(A)**, Gabra2 **(C)**, and Gabra4 **(E)**. **(B,D,F)** Quantification of percentage of MSN cells (dapi+ nuclei) expressing Gabra1 **(B)**, Gabra2 **(D)**, and Gabra4 **(F)**. ****p < 0.0001.

The overall proportion of Gabra1 expressing cells shows a trend toward a steady increase from P2 to P16, with approximately 80% of all NAc cells expressing Gabra1 by P16 (Figures 2A,B). While the results of the ANOVA indicate significant inter-group differences between the developmental timepoints, the difference between no two timepoints were found to be statistically significant in *post hoc* tests (Table 1, lower section). While there might not be a change in the proportion of cells that express Gabra1, the possibility remains that the level of expression within each cell changes over time. To delineate this, we investigated the number of Gabra1 transcripts per cell (Supplementary Figure S3A), which appeared unchanged between the three developmental time points. As noted, developmental changes in the expression of GABA_A_R subtypes are often quite similar across species in hippocampal and cortical regions. To investigate whether this might also be true for the NAc, we analyzed GABRA1 transcript expression in the human striatum using the Brainspan Atlas of the Developing Brain (Allen Institute for Brain Science, 2010) dataset. GABRA1 expression in the human striatum follows a slightly increasing trend from prenatal to early postnatal developmental period, similar to the trend observed in mice in the current study (Supplementary Figure S4A).

The proportion of cells expressing Gabra2 remained stable across the queried developmental timepoints, with approximately 85% of all NAc cells expressing Gabra2 (Figures 2C,D and Table 1). There was also no statistically significant change in mean number of Gabra2 transcripts expressed in NAc cells, although a nonsignificant increasing trend was detected on P16 (Supplementary Figure S3B). The human striatal data showed a similar pattern of mostly stable expression with a minor increasing trend (Supplementary Figure S4B).

There was a significant increase in the proportion of NAc cells expressing Gabra4, with significantly more cells expressing Gabra4 at P9 and P16 compared to P2 (Figures 2E,F and Table 1). While only about half of NAc cells expressed Gabra4 at P2, almost 95% of NAc cells were found to express Gabra4 by P16. In addition to the increase in the overall proportion of NAc cells expressing Gabra4, the number of transcripts observed in each cell also increased significantly from P2 to P9 to P16 (Supplementary Figure S3C). The expression of GABRA4 in the human striatum also followed a starkly increasing trend from prenatal through early postnatal development (Supplementary Figure S4C), supporting the conservation of GABA_A_R subunit expression changes across species.

### 3.3 Developmental changes in Gabra1 expression in different NAc MSN populations

**Figure 3:**
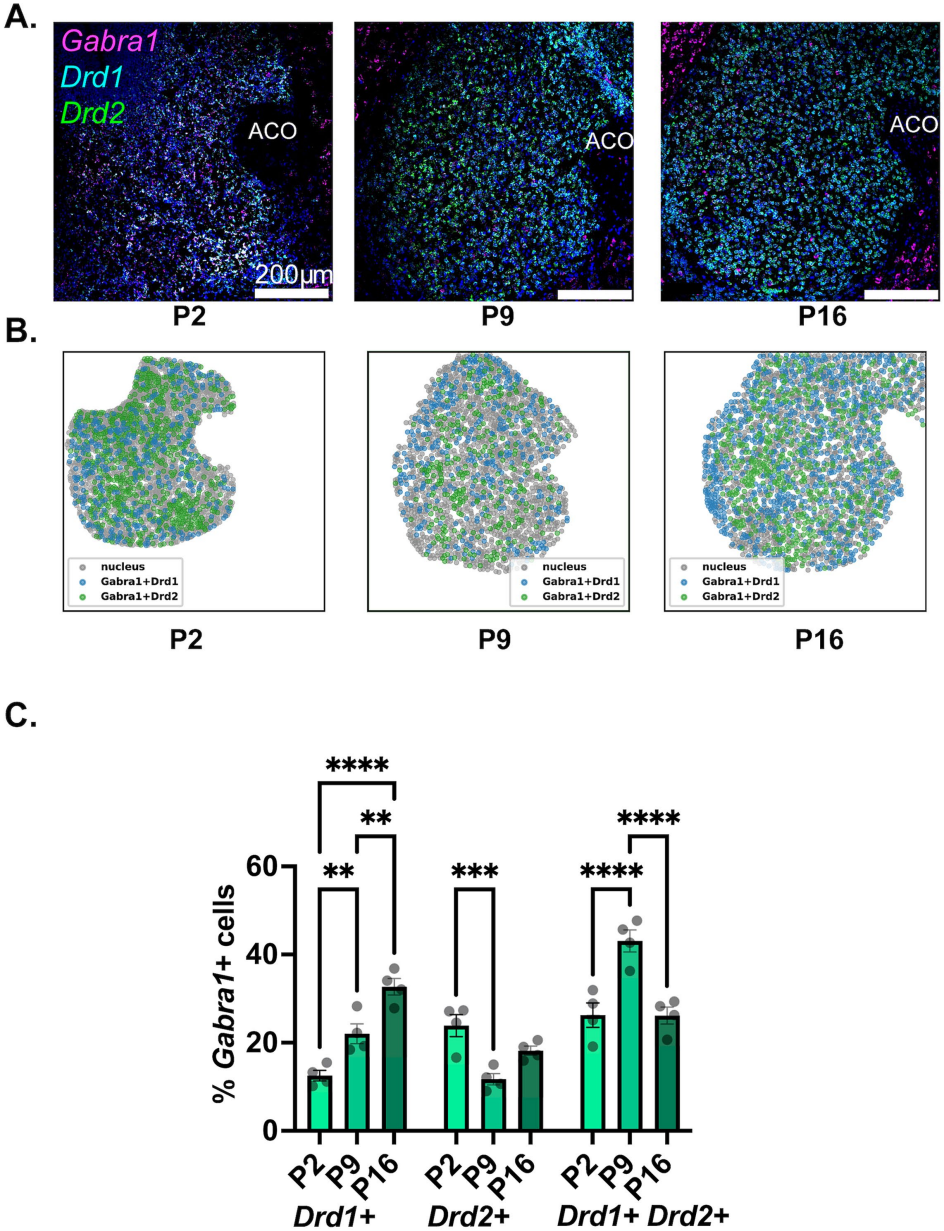
Developmental changes in the NAc cell-type specific expression of Gabra1. **(A)** Representative sections from P2, P9, and P16 mice probed for Drd1 (cyan), Drd2 (green), and Gabra1 (magenta). **(B)** Spatial density plots showing the distribution of Gabra1 expressing Drd1+ and Drd2+ MSNs. **(C)** Quantification of the percentage of Gabra1 expressing Drd1+, Drd2+, and Drd1+/Drd2+ NAc neurons across the three studied developmental timepoints. **p < 0.01, ***p < 0.001, ****p < 0.0001.

The findings regarding the developmental expression of Gabra1 in NAc MSN populations are depicted in Figure 3. Please note that in Section 3.1 above, we presented findings regarding the overall expression of Drd1 and Drd2, whereas here, we are using the presence of Drd1 or Drd2 in a given cell as a population marker for that MSN population. As such, in the graphs in this and the below two sections, Drd1+ MSNs represent cells that carry Drd1, but not Drd2, and vice versa. We are, in addition, presenting the Drd1+/Drd2+ population as a putative approximation to the cell type that has recently been deemed atypical or hybrid MSNs (Gagnon et al., 2017; Saunders et al., 2018; Stanley et al., 2020; He et al., 2021).

As seen in Figure 3C and is also visible in the representative confocal microscopy images in Figure 3A and in the spatial density graphs in Figure 3B, there is a significant and steady increase in the proportion of Drd1+ MSNs expressing Gabra1 from P2 to P9 to P16 (see Table 2, top panel for a summary of statistical comparisons). In contrast, in Drd2+ MSNs, there is a significant decrease in the proportion of Gabra1 expressing cells from P2 to P9, followed by a moderate increase by the P16 timepoint, such that the proportion of Gabra1+ Drd2+ MSNs at P16 is not significantly different from the proportion at P2 (Table 2). While at P2, approximately 12% of Drd1+ MSNs and 24% of Drd2+ MSNs express Gabra1, by P16, the situation is reversed, with over 30% of Drd1+ MSNs but less than 20% of Drd2+ MSNs expressing Gabra1.

The putative hybrid MSN population, Drd1+/Drd2+ cells, shows a stark increase in the proportion of Gabra1 expressing cells from P2 to P9, followed by a decrease back to P2 levels by P16 (Table 2). While at P2, this group has the highest proportion of Gabra1 expression out of the three MSN populations investigated, by P16, the Gabra1 expression lies halfway between the Drd1+ and Drd2+ populations.

### 3.4 Developmental changes in Gabra2 expression in different NAc MSN populations

**Figure 4:**
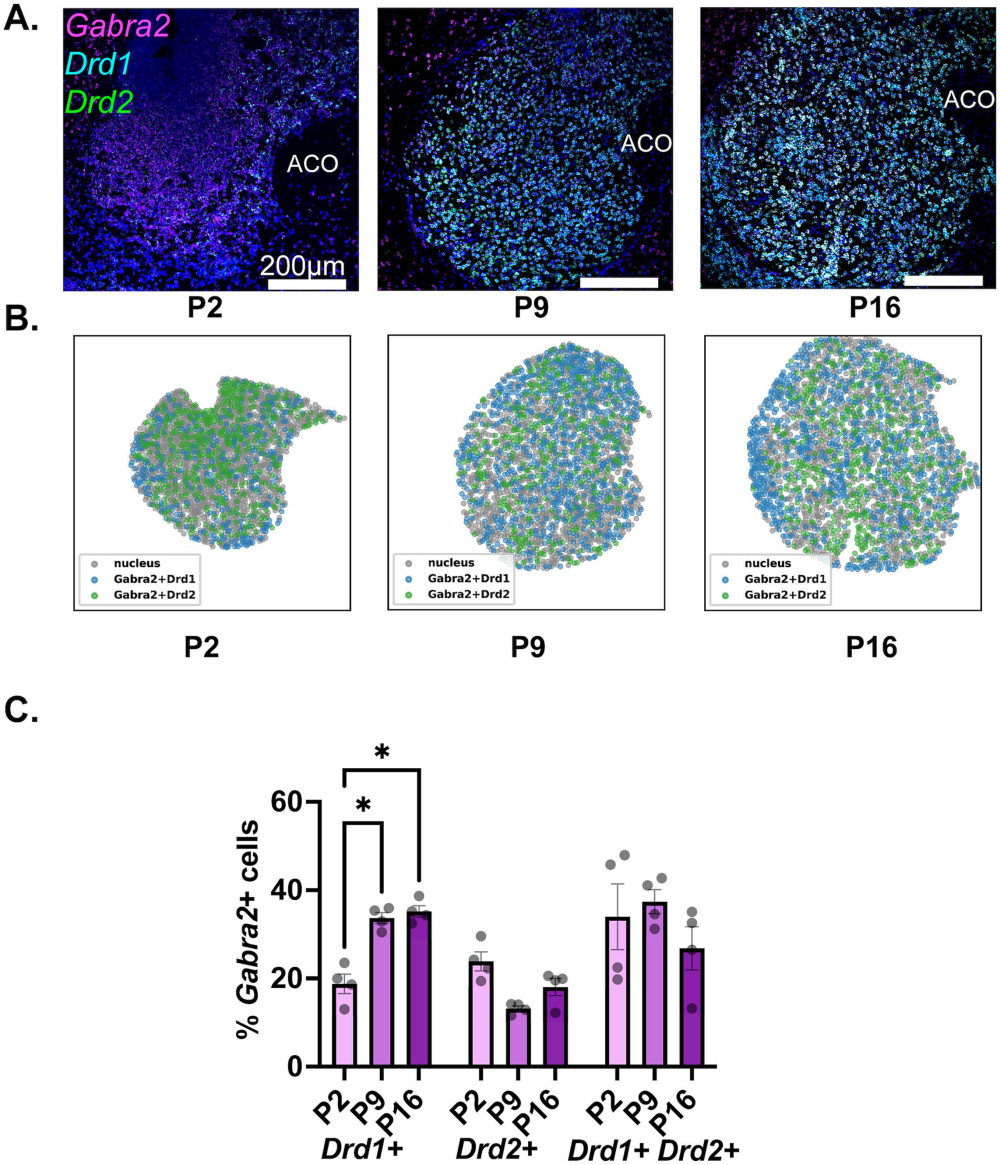
Developmental changes in the NAc cell-type specific expression of Gabra2. **(A)** Representative sections from P2, P9, and P16 mice probed for Drd1 (cyan), Drd2 (green), and Gabra2 (magenta). **(B)** Spatial density plots showing the distribution of Gabra2 expressing Drd1+ and Drd2+ MSNs. **(C)** Quantification of the percentage of Gabra2 expressing Drd1+, Drd2+, and Drd1+/Drd2+ NAc neurons across the three studied developmental timepoints. *p < 0.05.

The findings regarding the developmental expression of Gabra2 in NAc MSN populations are depicted in Figure 4. A significant increase in the proportion of Drd1+ MSNs expressing Gabra2 was observed from P2 to P9 (Figures 4A,B, left 2 panels; Figure 4C and Table 2, middle panel for a summary of the results of statistical comparisons). No further increase was observed from P9 to P16, with the proportion of Gabra2 expressing MSNs remaining around 36%. While a decreasing trend was observed in the proportion of Gabra2 expressing Drd2+ MSNs from P2 to P9, neither this difference nor changes between P9 and P16 were statistically significant (Figure 4C and Table 2, middle panel). At P16, approximately 20% of Drd2+ MSNs expressed Gabra2. Finally, there was no significant change in the proportion of Gabra2 expressing cells in the putative Drd1+/Drd2+ MSN population (Table 2).

### 3.5 Developmental changes in Gabra4 expression in different NAc MSN populations

**Figure 5:**
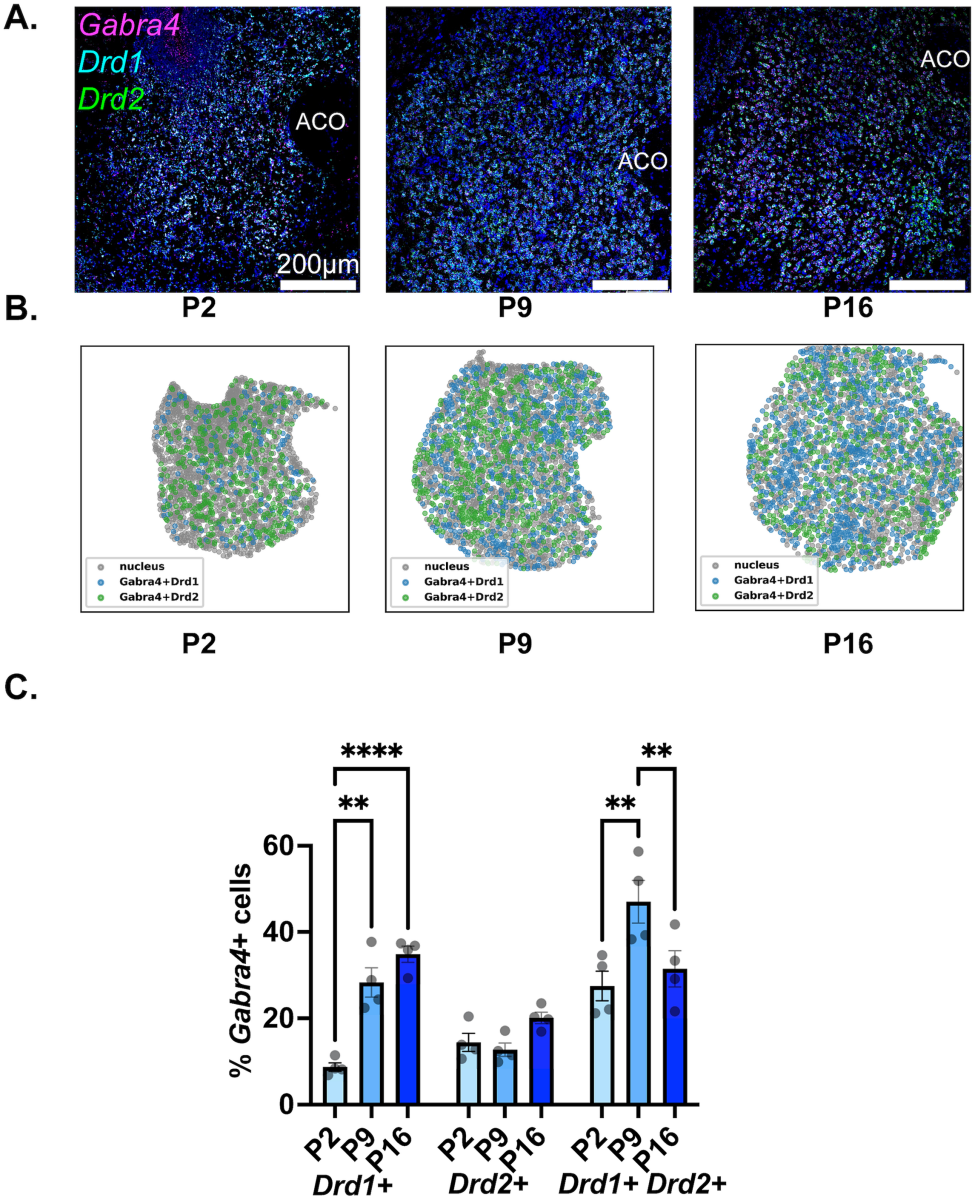
Developmental changes in the NAc cell-type specific expression of Gabra4. **(A)** Representative sections from P2, P9, and P16 mice probed for Drd1 (cyan), Drd2 (green), and Gabra4 (magenta). **(B)** Spatial density plots showing the distribution of Gabra4 expressing Drd1+ and Drd2+ MSNs. **(C)** Quantification of the percentage of Gabra4 expressing Drd1+, Drd2+, and Drd1+/Drd2+ NAc neurons across the three studied developmental timepoints. **p < 0.01, ****p < 0.0001.

The findings regarding the developmental expression of Gabra4 in NAc MSN populations are depicted in Figure 5. As seen in the representative confocal images (Figure 5A) and spatial density plots (Figure 5B), as well as the quantitative summary graph (Figure 5C), there was a steady and significant increase in the proportion of Drd1+ MSNs expressing Gabra4 from P2 to P9 to P16 (see Table 2, lower panel for a summary of the results of statistical comparisons). While less than 10% of Drd1+ MSNs expressed Gabra4 on P2, this proportion was close to 40% by P16. In contrast, the proportion of Gabra4 expressing Drd2+ MSNs remained relatively stable over time with no significant differences between the three investigated timepoints and with approximately 20% of Drd2 MSNs expressing Gabra4 (Table 2, lower panel). A similar pattern to Gabra1 was observed in the Drd1+/Drd2+ MSN population, with a significant increase in the Gabra4+ subpopulation from P2 to P9, followed by a decrease by P16 to bring it back to P2 levels (Table 2, lower panel).

## 4 Discussion

With this work, we aimed to address a significant gap in our knowledge of cell-type-specific changes in the expression of three prominently expressed GABA_A_R subtypes in the NAc during early postnatal development. Our findings in C57Bl/6 J mice suggest a small increase in overall Gabra1 expression, relatively stable overall Gabra2 expression, and a stark increase in overall Gabra4 expression over the 3 timepoints spanning P2 to P16. The overall increase in the percentage of NAc cells expressing Gabra1 and Gabra4 is in line with previous findings that the proportion of MSNs exhibiting spontaneous GABA_A_R-mediated currents increases steadily between P2 and P16 (Dehorter et al., 2011). This pattern of changes is also similar to earlier observations in the developing rat caudate putamen (Laurie et al., 1992). Analysis of data from the Brainspan dataset (Allen Institute for Brain Science, 2010) presented in Supplementary Figure S4 indicates similar trends in the expression of Gabra1, Gabra2, and Gabra4 in the human striatum in a period spanning prenatal and early postnatal development. While the differences in the developmental period covered in our studies and this data set are undeniable, the striking similarities in the expression trends suggest cross-species conservation of early developmental changes.

When investigated at an MSN-type-specific level, we see a stark increase in Drd1+ MSNs that express Gabra1, accompanied by a modest decrease in the proportion of Drd2+ MSNs expressing Gabra1. A similar pattern was observed with Gabra2 and Gabra4, where the proportion of Drd1+ MSNs expressing either GABA_A_R subunit RNA increased steadily from P2 to P16, whereas the proportion of Drd2+ MSNs expressing these subunits either remained stable or declined modestly over the same period. By P16, for all three investigated GABA_A_R subtypes, the proportion of Drd1+ MSNs expressing the subunit RNA is larger than the Drd2+ MSNs expressing the same subunit RNA. This finding is in line with previous reports that Drd1+ MSNs on average are less excitable than Drd2+ MSNs (Day et al., 2008; Gertler et al., 2008).

An interesting observation in several of our findings is the nonlinear nature of the changes where a stark change from P2 to P9 is followed by a leveling-off or even a slight reversal of the trend from P9 to P16. This nonlinearity could reflect developmental changes taking place between P9 and P16 that require different GABA_A_R-mediated regulation of MSN activity after P9 compared to before P9. One such significant change, the developmental GABA switch, is commonly considered to happen around P10 in rodents, although there is accumulating evidence suggesting that the exact timing of the switch is brain region, cell-type, and sex-dependent (Nunez and McCarthy, 2007; Murguia-Castillo et al., 2013; Murata and Colonnese, 2020). As activation of the studied GABA_A_Rs would lead to depolarization before the switch and hyperpolarization after, a shift in GABA polarity around P10 could explain the nonlinearity of GABA_A_R expression changes. However, GABA can have depolarizing effects even in adult MSNs, specifically when the MSNs are in a hyperpolarized “down” state, going against the idea of a clear switch from depolarizing to hyperpolarizing GABA around P10 (Tepper et al., 1998; Day et al., 2024). Still, the depolarizing effect of GABA was found to be larger in P2 compared to P30 MSNs, even in resting state (Dehorter et al., 2011). It is possible that there is inherent nonlinearity in this gradual decline in depolarizing efficacy which might overlap with the nonlinearity of GABA_A_R expression changes. Moreover, as glutamatergic synapses start developing in the NAc after P10, GABA loses its status as the sole depolarizing neurotransmitter in the NAc (Peixoto and Kozorovitskiy, 2020). The maturation of local glutamatergic neurotransmission between P9 and P16 could further contribute to the nonlinearity in GABA_A_R expression changes.

While our findings clearly indicate differential developmental regulation of specific GABA_A_R subtypes, it is difficult to ascertain the physiological roles of each receptor subtype at this stage. In the adult ventral striatum, at least during the up states, α1GABA_A_Rs and α2GABA_A_Rs mediate synaptic phasic inhibition, while α4GABA_A_Rs are expressed extrasynaptically and mediate tonic inhibition, contributing to the low excitability of MSNs (Maguire et al., 2014). Prior to the development of parvalbumin positive (PV+) interneuron synapses on MSNs around P9, it is possible that α1GABA_A_Rs and α2GABA_A_Rs also display a more extrasynaptic expression pattern and mediate tonic currents and that the increase in the percentage of Drd1+ MSNs expressing these receptors observed starting P9 could accompany their relocation to the increasing number of synapses made by PV+ interneurons on Drd1+ MSNs.

In addition to Drd1+ and Drd2+ MSNs, we are reporting findings from a group of neurons that express both Drd1 and Drd2. Recent studies suggest that these neurons likely represent a distinct class with different neuroanatomical properties, more dense expression in the ventral than dorsal striatum, particularly in the NAc shell, and different response to dopaminergic denervation than Drd1+ or Drd2+ MSNs (Gagnon et al., 2017). Putative Drd1/Drd2+ MSNs in our studies also showed a distinct pattern of changes in the expression of different GABA_A_R subunit RNA, with a stark increase in the percentage of cells expressing Gabra1 or Gabra4 from P2 to P9, followed by a decrease for both subunits. The percentage of putative Drd1+/Drd2+ MSNs expressing Gabra2, on the other hand, remained relatively stable around 30% during this time. Drd1+/Drd2+ striatal neurons have fewer dendritic spines, possibly suggesting reduced glutamatergic innervation compared to Drd1+ or Drd2+ MSNs (Gagnon et al., 2017). Our findings suggest that a larger proportion of these neurons express GABA_A_R subunits. Depending on the membrane characteristics, these neurons might represent a subclass that is less excitable than other MSNs and is highly protected against excitotoxic damage through extensive GABA_A_R expression.

The shell and core subdivisions of the NAc have different connectivity and serve different behavioral functions in adult animals (Groenewegen et al., 1999; Ambroggi et al., 2011; Dutta et al., 2021). It is, therefore, tempting to hypothesize different developmental patterns for the GABA system in these two subregions. As seen in the spatial density plots provided in Figures 3B, 4B, 5B, however, the three subunits investigated in this study all showed anatomically homogenous expression throughout the NAc with no clear accumulation in the shell or core subdivisions. Because of the visual homogeneity of expression, all quantitative analyses were conducted by combining the NAc shell and core.

The exact processes that lead to the developmental changes in GABA_A_R subunit expression are not known, however, evidence suggests a complex interplay of transcriptional, post-transcriptional, activity-dependent, and physiological/environmental factors. Inhibitory synaptogenesis leads to accumulation of GABA_A_Rs at synaptic sites, leading to an overall shift from high affinity GABA_A_Rs to lower affinity GABA_A_Rs whose properties are more suitable for the kinetics of phasic synaptic activity (Farrant and Nusser, 2005). Formation of new inhibitory synapses and maintenance of functional synapses is an activity-dependent processes that involves participation of several players including glutamate receptors (Oh and Smith, 2019). As noted above, protracted neurogenesis of inhibitory interneurons can also lead to changes in the source of GABA for both GABAergic synapses and the ambient GABA levels in extracellular space, affecting the levels of surface GABA_A_Rs. At least in adults, GABA_A_R subunit expression and GABA_A_R activity are also regulated by post-transcriptional (Steiger and Russek, 2004) factors and changes in receptor assembly and trafficking (Kittler and Moss, 2003), which could play early developmental roles in determining subunit expression levels.

While our studies provide novel insights into the early development of the GABA system in the ventral striatum, a few caveats should be noted. First, the studies are far from being a comprehensive examination of GABA_A_Rs across early development. As noted in the introduction, we focused on the *α*-subunit of the GABA_A_Rs due to the common approach of categorizing GABA_A_Rs into subtypes based on α subunit expression. We have chosen to focus on Gabra1, Gabra2, and Gabra4 due to their predominant expression in the adult striatum (Fritschy and Mohler, 1995; Hortnagl et al., 2013). However, studies suggest that a5GABA_A_Rs contribute to tonic GABA conductance in adult D2 MSNs (Santhakumar et al., 2010). Moreover, there is some evidence that Gabra3 might be expressed at moderate levels shortly after birth, reducing to its ultimate very low striatal expression within the first postnatal week in rats (Laurie et al., 1992) and others reported some weak Gabra3 labelling in NAc shell even in adult mice (Hortnagl et al., 2013). Thus, while our studies focus on the main mediators of GABAergic inhibition on MSNs, they do not provide a complete picture. Second, as noted in the Methods section, our studies employed tissue from male mice only. As most earlier studies we have referred and compared our results to throughout the manuscript were also conducted using tissue from male animals (e.g., Laurie et al., 1992; Fritschy and Mohler, 1995; Pirker et al., 2000; Hortnagl et al., 2013), this provided an opportunity to build on existing knowledge and expand the findings from previous work. However, it is possible that early developmental sex differences exist in the expression changes in GABA_A_Rs and this question should be addressed in future work. Finally, while we have chosen to focus on a time period where similar GABA_A_R expression changes are observed throughout the brain, studies suggest that MSNs continue to mature and changes in GABA_A_R expression patterns persist well into adolescence (Tepper et al., 1998; Santhakumar et al., 2010; Peixoto and Kozorovitskiy, 2020). Thus, future studies investigating GABA_A_R expression changes up to P30 might uncover further developmental patterns not covered by the current work.

While our findings suggest a dynamic landscape of cell-type-specific changes in GABA_A_Rs during early development, it is not clear what specific roles these changes play in development. There is, however, some evidence that developmental insults occurring during the period covered in this study can have long-lasting effects on the GABA_A_Rs of the NAc with significant behavioral consequences. Inspired by earlier findings that GABRA2 haplotypes are associated with cocaine addiction but only in individuals with backgrounds of childhood trauma (Enoch et al., 2010), Mitchell et al. (2018) exposed mice to an early life adversity (ELA) model between P2 and P9 and compared adult mice with ELA background to controls. They reported that MSNs of adult ELA mice showed reduced expression of Gabra2, with no change in Gabra1 or Gabra4 expression. The ELA mice showed increased locomotor effects of cocaine and reduced cocaine sensitization, similar to mice lacking the GABA_A_R α2 subunit (Dixon et al., 2014), suggesting that the reduction in NAc Gabra2 expression contributes to the changed response of ELA mice to cocaine. Unfortunately, the Mitchell et al. study did not investigate the change in Gabra2 expression or the reduction in phasic inhibition in the MSNs of ELA exposed mice in a cell-type specific manner. Our studies show that between P2 and P9, which is the time of ELA exposure in the Mitchell et al. study, the percentage of D1+ MSNs that express Gabra 2 almost doubles. It is possible that the exposure to stress during this time prevents this increase in Gabra2 expression in D1+ MSNs, leading to a hyperexcitability phenotype in D1+ MSNs. It is also possible that the modest decreasing trend in the proportion of Drd2+ MSNs expressing Gabra2 is potentiated by ELA exposure. Indeed, we previously reported that a D2+ MSN-selective knockdown of a2GABA_A_Rs leads to increased stress susceptibility in adult mice (Benham et al., 2021), providing a potential link between the experience of stress during early development and later stress susceptibility. Overall, our findings regarding dynamic and cell-type specific changes in the expression of different GABA_A_R subunits in the developing NAc provide a basis for understanding the effects of early life experience in NAc circuits and their behavioral consequences.

## Data Availability

The raw data supporting the conclusions of this article will be made available by the authors, without undue reservation.
