# Supplementary figures and images for: Early developmental changes in GABAA receptor expression in nucleus accumbens medium spiny neurons
Source: Front Neurosci. 2024 Dec 12;18:1445162. doi: 10.3389/fnins.2024.1445162 (PMC11669658; doi:10.3389/fnins.2024.1445162)

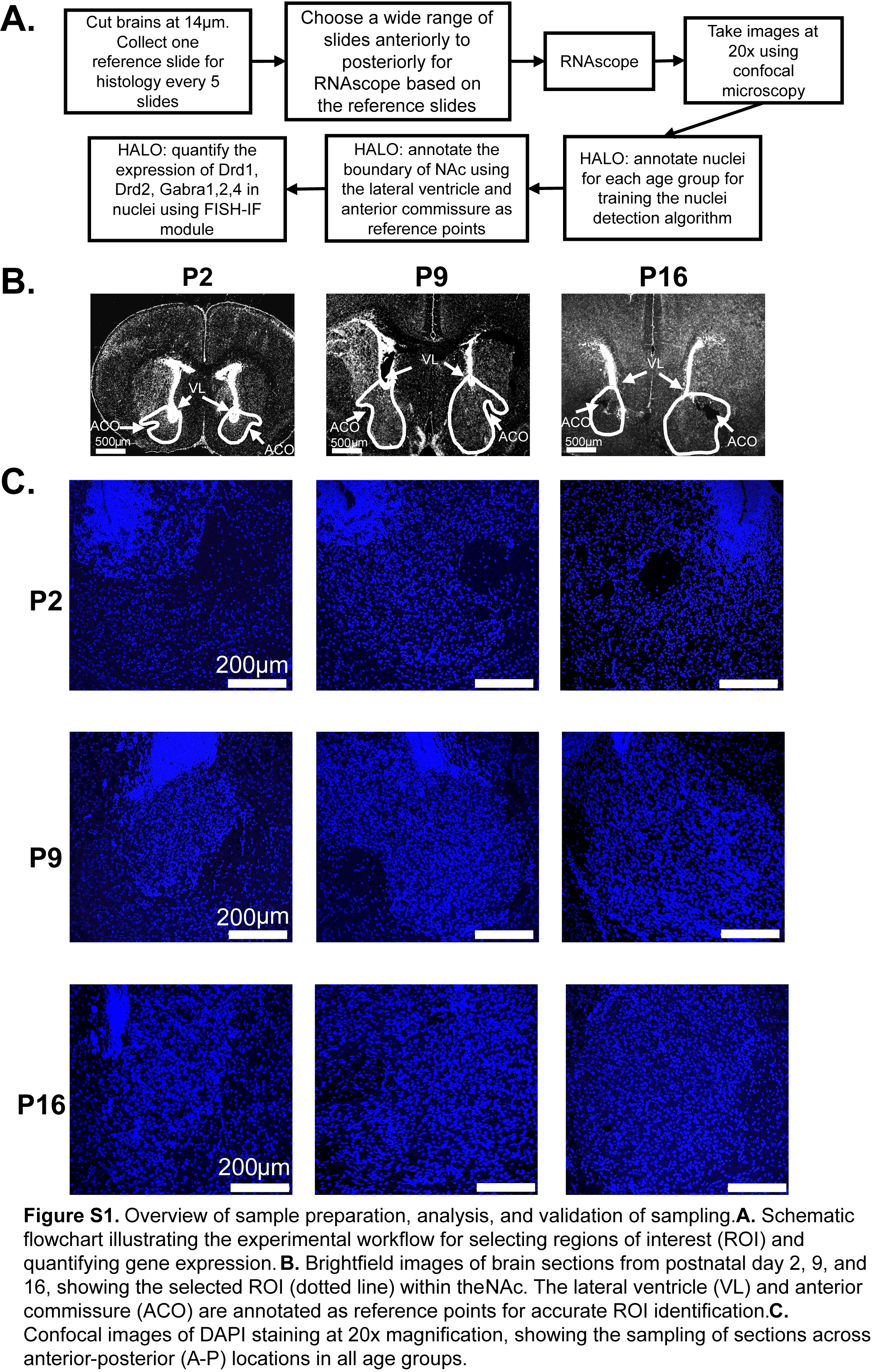

Supplement: Supplementary file 1 [file Image_1.TIF]

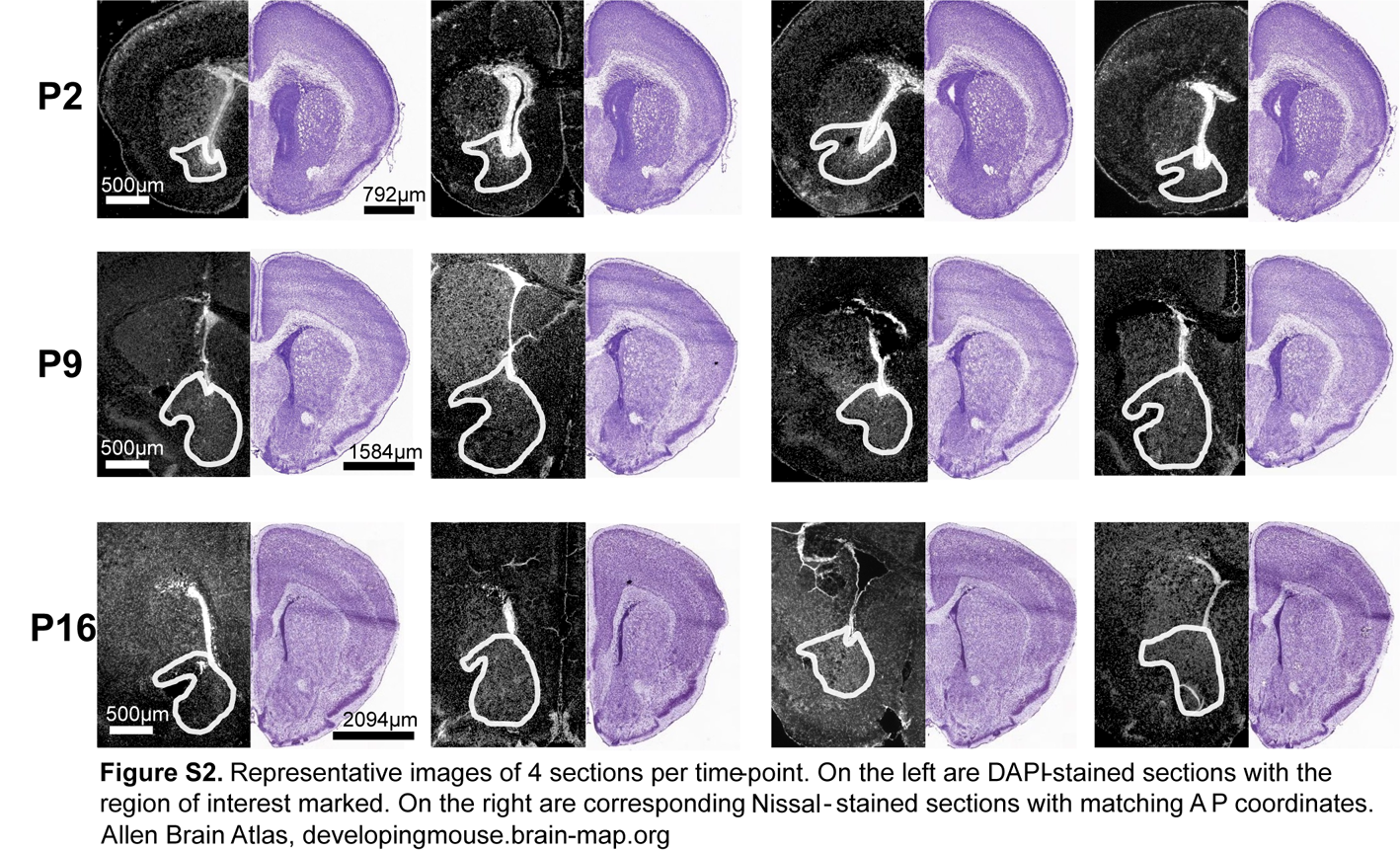

Supplement: Supplementary file 2 [file Image_2.tif]

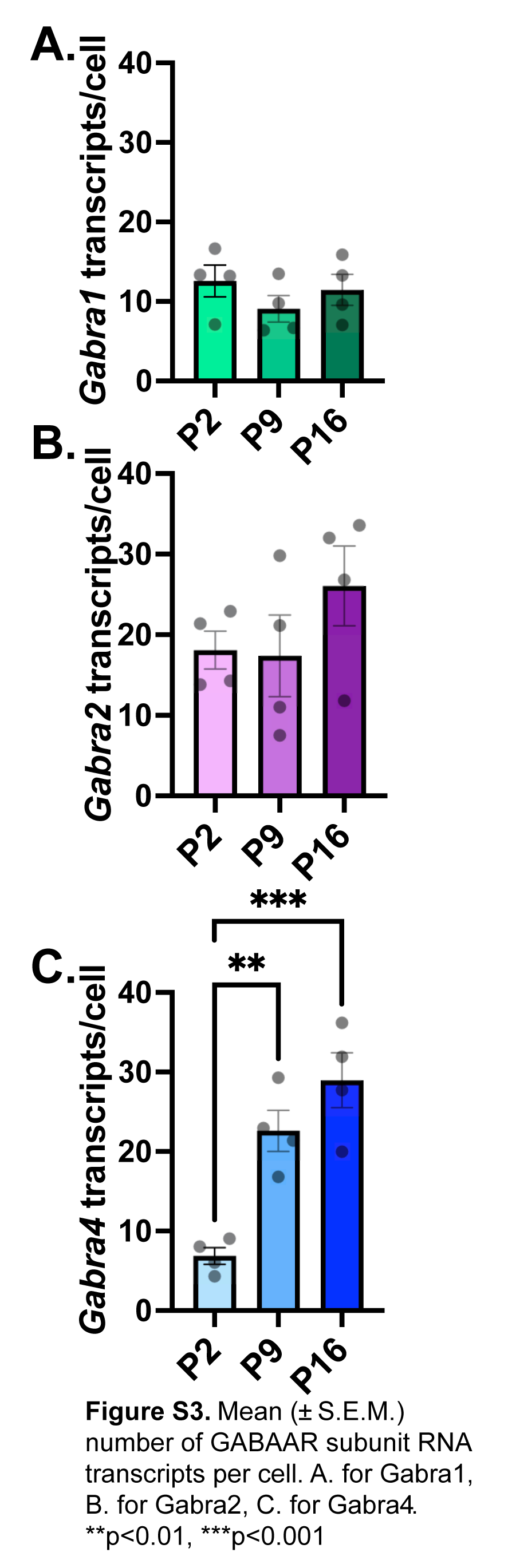

Supplement: Supplementary file 3 [file Image_3.TIF]

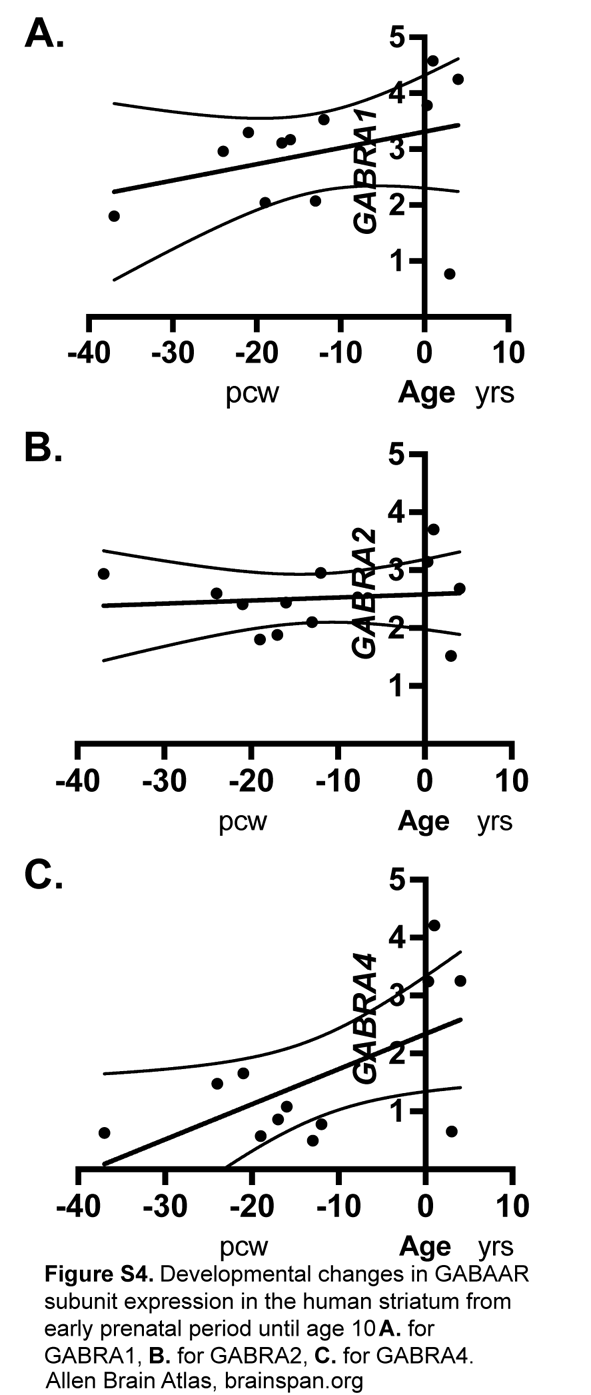

Supplement: Supplementary file 4 [file Image_4.tif]
